# Media Reporting of Affordable Cancer Care in High Income Countries: a Lancet Oncology Commission

**DOI:** 10.3332/ecancer.2011.ed12

**Published:** 2011-10-03

**Authors:** Richard Sullivan

**Affiliations:** Kings College, London, UK

## Lies, damned lies and truth.

Money and cancer care were always going to be a volatile combination for this Commission (***Lancet Oncology*** 2011 12(10): 923-980). Add in a complex subject, a lack of an educated (on the whole) media as well as strong ideological leanings and critical fusion was soon reached! One of the first phenomena to emerge was the Chinese whisper. In these days of e-media misinformation is rapidly passed on. In this case it started with the Daily Telegraph trumpeting that the conclusions were that we should deny dying patients beneficial treatments. Their strap-line ‘Dying cancer patients should not be given ‘futile’ drugs’ was pure fiction, although a lot of the text accompanying it was correct (a sizeable amount though was not). This theme rapidly caught on infecting about 40% of the subsequent e-media. To make matters worse a Daily Telegraph bloggist then repeated and even expanded on the more outlandish and frankly false ‘facts’. Remarkably the viral dissemination didn’t stop at e-media but managed to jump the ‘species’ barrier to get into radio. And not just any old radio but the BBC. From main to regional programs the ‘story’ was the same; doctors want to deny dying cancer patients beneficial treatment. What also emerged, however, from the radio interviews were two other serious issues. The first was an almost complete over-focus on medicines, to the point that many discussants honestly believed that cancer medicines were the key technology in the control and cure of cancer. This wasn’t a complete surprise. Some time ago we conducted a study of BBC website reporting of cancer; drug stories were the dominant feature by far (***Brit J Cancer*** 2008, 99: 569-76). What was a surprise was the inability of people to accept that this ‘reality’ was not true. More broadly there was an almost zero level of understanding of any of the drivers and issues to delivering affordable cancer care. The second issue was more ideological. This came through on both the responses to e-media and the radio interviews. No value can be put on a human life. To put it another way a substantial number (around 40% by my reckoning) said that we should pay for treatment or interventions no matter what the cost nor how little benefit the interventions could potentially give. The reality that nearly everything we do in life is framed within the economics of value of a statistical life was utterly rejected. Moreover the ability to see a societal duty, that these decisions had consequences and impact on other lives was given no importance. Social justice was about ‘me’. Returning for a moment to the ‘why’ behind the almost exclusive focus on cancer medicines it is clear that the professional-industrial-media complex must take full responsibility. Fed on a daily diet of drugs, if you’ll excuse the pun, is it any wonder about the huge misperceptions within the general public. Healthcare professionals and research funding organisations haven’t done enough to educate or, for that matter put out a balanced view on what really controls and cures cancer. And this has been driven along by the commercial reality of the pharmaceutical sector needing to create brand visibility. We have been seduced by the scientific and commercial zeitgeist of cancer medicines, and somewhere along the line we forgot our duty to explain that it’s the broad church of cancer – from basic science to psychosocial oncology - that is collectively responsible for cancer public health.

But lets not cry ‘doom’ too soon! There were some good radio debates, e.g. Radio 5 Live, who were able to see the wood from the trees. Was there a magic formula? Well they seemed to have done two things. The first was to give the debate time to evolve (avoiding the usual hyperactive bunny approach to news) and the second was to have a balanced group to really talk out and through the issues. Likewise on the e-media there were stunning islands of excellence. And from sources you would least expect – the Carrick Times for example was nearly a masterpiece (sorry…one tiny error….the Commission was peer reviewed!). One could also respect where the opinion might not be ones own, e.g. the Wall Street’s view that this Commission showed the ‘Obamacare’ was a socialist threat, but nevertheless had taken an ***informed*** perspective. The Independent also ran with a major and well informed piece the following day, although it too focused on just cancer medicines it was nevertheless well informed. What separated the wheat from the chaff was simply that the writers had bothered to read the Commission and / or press releases and / or watched the media launch which was publicly available on the web (http://www.ecancermedicalscience.com/tv/?play=1100&cid=0&scid=0&q=)

So what conclusions and solutions can we draw? Should we conclude that this is simply too complex an issue for the public and withdraw behind policy smoke-screens. Absolutely not! In some ways this is a wake up call. We have to stop hyping cancer and be honest about what the real issues and problems are. So here are my top three messages….

1. **Media-public is over-focused on cancer medicines:** Research funders, cancer centres and healthcare professionals need to re-balance the cancer score-card. With over 767 major patient organisations across Europe there is ample opportunity for real and tangible direct to public education. How we deal with media misrepresentation remains a hugely difficult task. Clearly in the e-media age we need to be more savvy than just simply press releases and briefings. E-campaigns are required to ensure the real facts get across.
2. **Life cannot have an economic value and the individual needs trump society:** One feels like the whole concept of the value of a statistical life is society’s dirty little secret. We don’t like to talk about this at all. Unfortunately when it comes to healthcare it will be essential. Somehow we need to close the moral and ethical circle with the economic one. Pretending it’s not there will not make it go away. More importantly is the need to get people to think like a society rather than just ‘me’. It doesn’t help that it’s the ‘me’ that is promoted and driven by the media. Healthcare is not an individual, one-way relationship, those who deliver and receive have equal and balancing duties and rights.
3. **Educate, educate, educate:** Public policy to deliver affordable cancer care cannot and should not be driven by reactionary media. But you’d be excused for thinking that that’s just the way it is now. Somehow we need to create the space, time and intelligence (not just information dump) for a really solid discussion, opinion and argument abut exactly what we, and by we I mean all the different ‘tribes’ – countries, patients, doctors etc., are prepared to do (and not to do).

Of course the debate on affordability goes much, much further all the way to the gates of healthcare provision. But you have to start somewhere and cancer is as good a place as any.

**End-note:** Here is the pick of the e-media from a truthfulness and accuracy perspective (from well over 300 articles….)

**BBC**

http://www.bbc.co.uk/news/health-15032862

**NHS Choices**

http://www.nhs.uk/news/2011/09September/Pages/cost-of-advanced-cancer-drugs-questioned.aspx

**Reuters**

http://in.reuters.com/article/2011/09/26/idINIndia-59553920110926

**Carrick Times**

http://www.carrickfergustimes.co.uk/news/health/costs_of_cancer_treatments_questioned_1_3095336

**Independent**

http://www.independent.co.uk/life-style/health-and-families/features/drugs-the-doctors-dilemma-2361871.html

**Med India**

http://www.medindia.net/news/Research-Says-Unequal-Access-to-Cancer-Care-can-No-Longer-be-Tolerated-91281-1.htm

**Top News (USA)**

http://topnews.us/content/243694-cancer-treatment-cost-rising-too-fast

**Third Age (USA)**

http://www.thirdage.com/news/cancer-cost-rising-becoming-unaffordable_09-26-2011

**TIME magazine (USA)**

http://healthland.time.com/2011/09/26/report-cost-of-cancer-is-becoming-unaffordable/

